# Obstructive Sleep Apnea, Metabolic Dysfunction, and Periodontitis—Machine Learning and Statistical Analyses of the Dental, Oral, Medical Epidemiological (DOME) Big Data Study

**DOI:** 10.3390/metabo13050595

**Published:** 2023-04-26

**Authors:** Noya Ytzhaik, Dorit Zur, Chen Goldstein, Galit Almoznino

**Affiliations:** 1 In Partial Fulfillment DMD Thesis, Faculty of Dental Medicine, Hebrew University of Jerusalem, Jerusalem 91120, Israel; 2 Medical Information Department, General Surgeon Headquarter, Medical Corps, 02149, Israel Defense Forces, Tel-Hashomer, Israel; 3 Faculty of Dental Medicine, Hebrew University of Jerusalem, Israel; Big Biomedical Data Research Laboratory; Dean’s Office, Hadassah Medical Center, Jerusalem 91120, Israel; 4 Faculty of Dental Medicine, Hebrew University of Jerusalem, Israel; Department of Endodontics, Hadassah Medical Center, Jerusalem 91120, Israel; 5 Faculty of Dental Medicine, Hebrew University of Jerusalem, Israel; Department of Oral Medicine, Sedation & Maxillofacial Imaging, Hadassah Medical Center, Jerusalem 91120, Israel

**Keywords:** obstructive sleep apnea (OSA), metabolic syndrome, periodontitis, electronic medical record, machine learning, algorithm, Big Data analysis

## Abstract

This study aimed to analyze the associations of obstructive sleep apnea (OSA) with dental parameters while controlling for socio-demographics, health-related habits, and each of the diseases comprising metabolic syndrome (MetS), its consequences, and related conditions. We analyzed data from the dental, oral, and medical epidemiological (DOME) cross-sectional records-based study that combines comprehensive socio-demographic, medical, and dental databases of a nationally representative sample of military personnel for one year. Analysis included statistical and machine learning models. The study included 132,529 subjects; of these, 318 (0.2%) were diagnosed with OSA. The following parameters maintained a statistically significant positive association with OSA in the multivariate binary logistic regression analysis (descending order from highest to lowest OR): obesity (OR = 3.104 (2.178–4.422)), male sex (OR = 2.41 (1.25–4.63)), periodontal disease (OR = 2.01 (1.38–2.91)), smoking (OR = 1.45 (1.05–1.99)), and age (OR = 1.143 (1.119–1.168)). Features importance generated by the XGBoost machine learning algorithm were age, obesity, and male sex (located on places 1–3), which are well-known risk factors of OSA, as well as periodontal disease (fourth place) and delivered dental fillings (fifth place). The Area Under Curve (AUC) of the model was 0.868 and the accuracy was 0.92. Altogether, the findings supported the main hypothesis of the study, which was that OSA is linked to dental morbidity, in particular to periodontitis. The findings highlight the need for dental evaluation as part of the workup of OSA patients and emphasizes the need for dental and general medical authorities to collaborate by exchanging knowledge about dental and systemic morbidities and their associations. The study also highlights the necessity for a comprehensive holistic risk management strategy that takes systemic and dental diseases into account.

## 1. Introduction

Obstructive sleep apnea (OSA) is a common chronic sleep-related breathing disorder characterized by repetitive episodic pharyngeal collapse of the upper airway during sleep [1,2]. This often results in periodic or partial reductions (causing hypopnea), or cessations (causing apnea) in ventilation, which cause hypoxia, hypercapnia, or arousals from sleep [1,2]. An apnea is classified by respiratory sensors and is defined by the complete absence or near complete absence of airflow for at least 10 s [3]. Subsequently, OSA often results in poor sleep quality which causes daytime fatigue and sleepiness, loud snoring, witnessed interrupted breathing during sleep, mood disturbances and morning headaches [4]. 

It has also been found that OSA may contribute as a risk factor for several other clinical conditions, including hypertension, cardiovascular disease, and abnormalities in glucose metabolism [5,6]. Indeed, metabolic syndrome (MetS), composed of insulin resistance, dyslipidemia, central obesity, hypertension and abnormal fasting glucose or diabetes, share remarkably similar risk factors to OSA [7,8,9,10].

OSA also shares numerous risk factors with dental problems, which include periodontitis and caries. Periodontitis, the most common chronic inflammatory non-communicable disease of humans, is a chronic inflammatory disease that causes a continuous destruction of the teeth-supporting tissues, such as the periodontal ligament and the alveolar bone [8,9,10]. Periodontitis is caused by complex interactions between pathogenic bacteria, ruinous host immune responses, and environmental risk factors such as smoking [8,9,10]. Similar to OSA, risk factors for periodontitis include smoking and diabetes mellitus [8,10]. 

Dental caries is the most common disease in the world and is caused by a dental biofilm of microorganisms that, in combination with other genetic and environmental factors, can generate tooth decay [11]. Dental caries also shares similar environmental risk factors to OSA, such as sugar-rich diet, smoking, bacteria, and inflammation [11]. A diet rich in carbohydrates or sugars could cause obesity and thus lead to caries and periodontitis. In addition, periodontitis and dental caries are both linked to inflammation, which is associated with OSA [12]. 

The existing literature regarding the possible associations between OSA and periodontitis and dental caries yields varied and contradictory findings. On the one hand, some studies had concluded that OSA has a direct and positive association with periodontitis [13,14,15,16], whereas other studies found no or insufficient evidence of a connection between OSA and periodontitis [17,18,19]. Similarly, some research had found an association between dental caries and OSA [20,21], whereas other research found no such connection between caries and OSA [22]. Such contradictory findings might be credited to the limits of the research literature, including different definitions of dental and systemic conditions and the existence of confounding factors that had not always been taken into account. 

Given these limitations, it is important to conduct large-scale research regarding dental status and OSA associations, that follow a strict protocol for dental and medical disease definitions and consider the presence of numerous confounding factors [23].

Moreover, whilst most research has been using only statistical methods to tackle the topic, machine learning (ML) methodologies in artificial intelligence have recently been utilized to choose the most important variables (namely the feature selection/feature importance) in the identification of caries [23], and periodontitis [24] including our publication which studied the associations between BMI and dental caries using ML and statistical models [25]. To the best of our knowledge, no previous research has been published using statistical and machine learning models to study the associations between OSA and dental status in the context of metabolic dysfunction. 

Considering these unmet needs, the primary aim of this study was to analyze the association between OSA and dental status. To account for potential confounding factors, we investigated the associations of OSA with dental parameters using a statistical and ML models that control for sociodemographic characteristics, general and dental health-related practices, medical and dental attendance patterns, and MetS-related diagnoses and auxiliary tests used in the assessment of MetS components.

The main research hypothesis is that an OSA diagnosis will be associated with worse dental status. This association will be reflected in higher periodontitis rates among people who have OSA and is based upon prior evidence of said connection through common inflammatory markers [13,14]. This will also come into fruition through more caries experience in OSA patients [20,21]. We also hypothesized that OSA will be associated with older age, male sex, and obesity, due to those associations being extensively documented in the existing literature [26,27]. 

## 2. Methods

### 2.1. Data Source

This study is a part of the Dental, Oral, Medical Epidemiological (DOME) record-based big data study [25,28,29,30,31,32,33,34]. These previous publications have used and described the DOME study, including a paper dedicated to the describe the protocol and study methods of the DOME [28]. The current paper compares socio-demographic dental, and medical records and examines their associations with OSA in a population of young to middle-aged military personnel who were actively serving in the Israel Defense Forces (IDF) in the period of 2015–2016. The IDF Medical Information Department provided all data from three IDF computerized systems: dental patient record (DPR), medical (i.e., computerized patient record (CPR), and socio-demographic computerized systems that store personal socio-demographic profiles of all military personnel, as described previously [28]. The current study is a cross-sectional big data study includes a unique national representative sample of young to middle-aged adults of military personnel with socio-demographic, dental and medical data, and provides us with the unique opportunity to cross these parameters with OSA diagnosis and thus analyze its associations with dental and oral conditions on a large and unmatched scale.

### 2.2. Ethical Approval

The study adheres to the STROBE (Strengthening the reporting of observational studies in epidemiology) guidelines. Approval from the Institutional Review Board had been granted (Medical Corps IRB number: 1281–2013). The data examined are anonymous and contain no identifying information. The IRB granted a dispensation from informed consent in writing because the study was retrospective and included anonymized record analyses. 

### 2.3. Criteria for Enrollment

Criteria for inclusion: The socio-demographics, health, and dental records of IDF members, men and women aged 18–50 years, who attended IDF Dental clinics between 1 January 2015 and 1 January 2016, and whose data exist in the socio-demographic medical, and dentistry military records.

Criteria for exclusion: Subjects with incomplete information within those data sources have been precluded from participation. 

### 2.4. Definition and Examination of the Variables

OSA was analyzed as the dependent variable and the other parameters served as the independent variables. The DOME protocol and study methods paper has previously specified the definitions of the variables available in the repository [28], and they will be briefly present them here.

#### 2.4.1. Sociodemographic Characteristics

Age and length of service: Continuous variables in years.

Sex: Dichotomous variable, nominal: men/women.

Educational level: Categorial variable with three categories: 1. high school graduate of twelve years at schooling. 2. technician certificate. 3. academic education.

Residency location: Categorial variable, nominal with three options: urban Jewish, urban non-Jewish, and rural.

Socio Economic Status (SES): Categorial variable, ordinal based on an ordinal scale of 10 deciles as determined by the Israeli Ministry of the Interior and aggregated into three categories: low (1st–4th deciles), medium (5th–7th), and high (8th–10th).

Rings of a city/town: Dichotomous variable, nominal: midtown: living in a core of a city/town vs. suburbs.

Countries of birth: Categorial variable, nominal: North America, Eastern Europe, Western Europe, Ethiopia, Africa, Asia, South America, and Israel.

#### 2.4.2. Health-Related Habits and Attendance Patterns

The following self-reported health-related lifestyle habits are listed as dichotomous variables, nominal (yes/no): current smoking, and daily tooth brushing (at least once a day). Attendance patterns, discrete variable included: total number of dental and medical appointments as well as non-attendance to scheduled dental appointments.

#### 2.4.3. Medical Diagnoses and Auxiliary Test Results Definitions

The auxiliary test results and medical diagnoses of MetS components, sequelae, and associated morbidities were obtained from the CPR in the manner previously described [28]. The CPR uses the International Classification of Diseases, Ninth Revision, Clinical Modification (ICD-9-CM) ICD-9-CM as the basis for its diagnoses. Diagnoses were dichotomous nominal variables (yes/no).

The dependent variable: Obstructive sleep apnea (OSA) is the dependent variable that corresponds to the 2015 015 ICD-9-CM Diagnosis Code 327.23 Obstructive sleep apnea (adult) (pediatric). OSA is diagnosed using a combination of clinical evaluation and objective testing, such as polysomnography (PSG) [35]. A diagnosis of OSA is made if the apnea-hypopnea index (AHI) is between 5- and 14-h sleep plus one or more sleep-associated conditions, such as sleepiness, fatigue, or insomnia, or if the AHI is ≥15/h sleep [35].

Other medical diagnoses and axillary tests related to MetS: Other medical diagnoses related to MetS served as independent variables and were also based on the ICD-9-CM, as previously mentioned [25,28,32,33,34], and include diabetes mellitus, impaired glucose tolerance (IGT), hyperlipidemia, hypertension, cardiovascular disease, obesity (BMI > 30 kg/m^2^), obstructive sleep apnea (OSA), S/P (status post) transient ischemic attack (TIA), and S/P stroke [28,32,34].

Auxiliary Test Results: Results were also obtained from the CPR included tests used in the evaluation of MetS components, as mentioned previously [28,32,34], and were continuous variables: weight (in kilograms), body mass index—BMI (weight/height^2^ (kg/m^2^)), C reactive protein-CRP (mg/L), glycated hemoglobin-HbA1c (%), fasting glucose (mg/dL), cholesterol (mg/dL), high-density lipoprotein-HDL (mg/dL), low-density lipoprotein-LDL (mg/dL), triglycerides (mg/dL), very-low-density lipoprotein-VLDL (mg/dL), and non-HDL cholesterol (mg/dL).

#### 2.4.4. Dental Parameters

Past publications including the DOME methods paper [28,32,33] provide more information on the standardized uniform codes used in the DPR for each dental procedure and diagnostic. In a summary, the dental codes in the DPR are equivalent to the current dental terminology (CDT) of the American Dental Association (ADA) [28]. The DPR was used to retrieve the delivered treatments (i.e., dental treatments that were carried out) which included: fillings, endodontic treatments and retreatments, regular and surgical extractions, direct and indirect post and core, and crowns [28]. In addition, records of periodontal disease and a count of missing teeth (apart from wisdom teeth) were also retrieved from the DPR and included in the study as described previously [28,34].

### 2.5. Methods of Statistical and Machine Learning Analytics

#### 2.5.1. Statistical Analyses

The IBM, Chicago, IL, USA, SPSS software, version 28.0, was used to conduct the statistical analyses.

Descriptive statistics. Continuous variables were displaced as means± standard deviations, and categorical variables were displaced frequencies and their percentages.

Univariate analysis. To examine the associations between OSA and the independent variables, we conducted a Pearson’s chi-squared (χ^2^) or likelihood ratio test for categorical parameters, and an independent *t*-test for continuous variables. Binary logistic regression analysis was used to determine odds ratio (OR) for categorical variables, while linear regression analysis was used to obtain OR for continuous variables.

Analysis of multicollinearity. After the univariate analyses, we used linear regression to run multicollinearity tests to look at the collinearity of the independent variables. Only one of the highly correlated variables was used in the multivariate model, and the context determined which variable would be used in the model. The variance inflation factors (VIFs), which are 1/tolerance, were determined. Although VIF 10 is frequently used as a marker for collinearity, the current study utilized VIF 2.5 as a limit since VIF > 2.5 may be concerning in weaker models.

Multivariate analysis. After the univariate analyses and collinearity statistics, a multivariate binary logistic regression analysis was carried out with OSA as the dependent variable, with statistically significant independent parameters that were not highly collinear in the univariate analysis.

#### 2.5.2. Machine Learning (ML) Models

To run machine learning models we used python scikit-learn package [36]. We utilized XGBoost, an effective gradient boosting framework for supervised machine learning for both regression and classification applications [37]. Using the same set of parameters that were utilized in the statistical models, we investigated the relative clinical feature importance of OSA as the target variable. With five-fold cross-validation, we have run the model with different ratios of training and testing datasets (for example, Train-Test: 70–30% and 80–20%).

Sensitivity analyses: To confirm the validity of the XGBoost ML model, we also ran two additional ML models for feature importance: Gini Importance [38] and Information Gain (using Entropy) [39]. Both methods produced results for the goodness-of-fit model measurements, such as area under the curve (AUC) and accuracy, that were remarkably similar to the XGBoost.

Adherence to reporting guidelines and standards in the field of machine learning.

The TRIPOD ((Transparent Reporting of a Multivariable Prediction Model for Individual Prognosis or Diagnosis; www.tripod-statement.org, accessed on 10 April 2023) checklist for prediction model validation was used to assess the completeness of the reporting of this research study. The checklist consists of 20 main items and a total of 31 sub-items, which cover various aspects of prediction model validation. The items on the checklist relate to the title, abstract, background, methods, results, discussion, supplementary material, and funding information of the study. Each included item was given a “1” for adherence and a “0” for nonadherence. The analysis revealed that the study adheres to all a TRIPOD items, with three items being non relevant. The outcomes of each TRIPOD item were meticulously documented.

## 3. Results

### 3.1. The Associations between OSA and Socio-Demographic Parameters

The study included 132,529 subjects; of these, 318 (0.2%) were diagnosed with OSA. Table 1 presents the associations between socio-demographic parameters and OSA among the study population. OSA diagnosis had a statistically significant positive association with the following parameters in the univariate analysis: male sex (odds Ratio (OR) and 95% confidence interval (CI) men/women = 5.52 (3.45–8.93)), rural residency location (OR rural/urban Jewish locality = 4.20 (1.86–9.48)), age (OR = 1.19 (1.18–1.21)), and length of service (OR = 1.09 (1.08–1.10)) (Table 1).

High school education was negatively associated with OSA compared with academic education (OR = 0.04 (0.03–0.05)] (Table 1).

There were no statically significant associations between OSA and: technical education (OR technical/academics = 1.19 (0.93–1.53)), urban non-Jewish (OR urban non-Jewish/urban Jewish= 0.70 (0.48–1.02)), socio economic status (SES) (OR high/low SES = 1.63 (0.83–3.19), OR medium/low SES = 1.47 (0.75–2.89)), rings of a city/town (midtown/ suburbs = 1.06 (0.74–1.53)) and birth countries compared with native Israelis as a reference (Table 1).

### 3.2. The Associations of OSA with Health-Related Habits and Attendance Patterns

Table 2 presents the associations of OSA with health-related habits, as well as the medical and dental attendance patterns.

Health-related habits: OSA was positively associated with current smoker status (OR = 10.75 (8.62–13.51)) and negatively associated with teeth brushing at least once a day (OR = 0.54 (0.41–0.73)) (Table 2). 

Attendance patterns: Patients with OSA, had more dental appointments (OR = 1.03 (1.02–1.03)), were more likely to not attend to scheduled dental appointments (OR = 1.06 (1.05–1.08)), and had more appointments with a general physician (OR = 1.02 (1.01–1.03)) (Table 2).

### 3.3. The Associations between OSA and Metabolic Morbidity

Table 3 presents the associations between OSA and MetS components, consequences, and associated illness among the study population. OSA was positively associated with the following conditions: hypertension (OR = 8.55 (6.45–11.36)), hyperlipidemia (OR = 5.85 (3.37–10.10)), diabetes mellitus (OR = 17.54 (10.42–29.41)), obesity (OR = 23.81 (18.87–29.41)), cardiovascular disease (OR = 7.63 (5.75–10.20)), non-alcoholic fatty liver disease (NAFLD) (OR = 17.24 (12.34–24.39)), stroke (OR = 9.09 (2.30–35.71)), and transient ischemic attack (TIA) (OR = 8.47 (2.14–33.33)) (Table 3). 

### 3.4. The Associations between OSA and Medical Indices

Table 4 presents the associations of OSA with the medical indices of the patients There were no statically significant associations between OSA and glycated hemoglobin (OR= 1.207 (0.967–1.505)) and fasting glucose (OR = 1.010 (0.989–1.032)). All other medical indices had a statistically significant positive, although weak associations with OSA, except for high-density lipoprotein (HDL) which had a statistically significant weak negative association with OSA (see Table 4).

### 3.5. The Associations between OSA and the Dental Status

Table 5 presents the associations of OSA with periodontitis, missing teeth and with the number of delivered dental procedures among the study population. OSA was positively associated with periodontal disease (OR = 3.46 (2.51–4.76)) and with the number of missing teeth (OR = 1.13 (1.09–1.18)). OSA also had a statistically significant positive association with all delivered dental procedures including fillings (1.05 (1.01–1.10)), endodontic treatments (OR = 1.43 (1.22–1.68)), regular extractions (OR = 1.19 (1.09–1.30)), surgical extractions (OR = 1.37 (1.07–1.75)), indirect post and core (OR = 1.38 (1.23–1.54)), direct post and core (OR = 1.59 (1.29–1.95)), crowns (OR = 1.25 (1.04–1.45)) and (Table 5).

### 3.6. Multivariate Analysis for OSA as the Dependent Variable

Following the univariate analyses, a linear regression analysis was performed to assess the collinearity between statistically significant the independent variables among the study population (Table 6). The results of collinearity statistics shown on Table 6 ruled out collinearity (VIF < 2.5). Subsequently, a multivariate binary logistic regression analysis was performed for OSA as the dependent variable (Table 6). The parameters were entered into the analysis simultaneously. The parameters that retained a statistically positive association with OSA in the multivariate analysis were the following (from the highest to lowest OR): obesity (OR = 3.10 (2.18–4.42)), male sex (OR = 2.41 (1.25–4.63)), periodontal disease (OR = 2.01 (1.38–2.91)), smoking (OR = 1.45 (1.05–1.99)), and age (OR = 1.14 (1.12–1.17)) (Table 6). 

### 3.7. Features Importance Based on XGBoost Machine Learning Algorithm with OSA Set as a Target Variable

**Figure 1:**
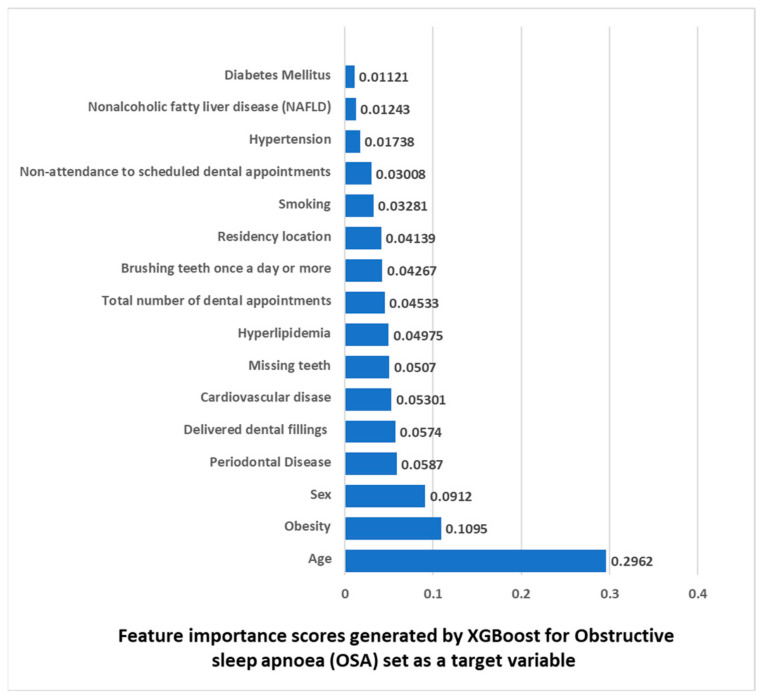
Feature importance scores generated by XGBoost for Obstructive sleep apnea (OSA) as a target variable.

The purpose of the XGBoost model shown in Figure 1 was to find the important features to predict OSA. The AUC was 0.868, and the accuracy of 0.927. The AUC of this model is considered an excellent discrimination. The results of the feature importance scores generated by XGBoost shown in Figure 1 are that among the highest ranked features that are increasing the risk of OSA there are well known risk factors for OSA of age, obesity, and sex (first–third places in feature importance), and following these features were periodontal disease (fourth place) and delivered dental fillings (fifth place). 

## 4. Discussion

A nationally representative sample of 132,529 young and middle-aged adults was used to examine the associations between OSA and dental parameters while adjusting for sociodemographic factors, health-related behaviors, and each MetS component, consequence, and related conditions. Even after the multivariate analysis, this big data study showed statistically significant associations between OSA and periodontal disease. Furthermore, we employed machine learning algorithms to support the statistical models. Figure 1 findings demonstrate that in addition to well-known risk factors for OSA, such as age, obesity, and sex (located on places 1–3) there were our also periodontal disease (fourth place) and delivered dental fillings (fifth place). Altogether, the findings supported the main hypothesis of the study, which was that OSA is linked to dental morbidity, in particular to periodontitis.

In the present study, OSA was found to be positively associated with age and being of the male sex, as was demonstrated in the multivariate analysis (Table 6) as well as in the ML model, which showed that age is in the first place and sex is in the third place in feature importance for ASA as a target variable (Figure 1). These results fall in line with previous studies, that have demonstrated an increase in OSA diagnosis with age for both men and women [27,40]. An increase in OSA prevalence was seen from young adulthood until the sixth and seventh decade, then appears to plateau [41]. A consensus also exists that men have higher OSA rates than women [27,40,42], a gap that decreases in postmenopausal [42]. Some mechanisms to explain this increase with age have been suggested before, such as lengthening of the soft palates with increasing age, increased deposits of fat in the parapharyngeal area, and changes in the tissues around the pharynx [43]. 

This current study found no significant association between SES and OSA. This conclusion is supported by other studies that found that there is no significant association between OSA and SES [44,45]. A systematic review by Etindele Sosso et al. disputed this claim and concluded that a link between lower SES and OSA exists, and questioned the approach used often in research into this topic, of including a single question to identify SES [46]. The advantage of our DOME study is that the SES variable is not based on a single question, since the SES variable is retrieved from the Ministry of Interior and is a validated score that considers age distribution, available workforce, level of unemployment, level of education, average per capita income, and proportion of income support recipients [28].

This current big data study found no significant association between OSA and ethnicity. In line, Hnin et al. also found that OSA severity and prevalence are comparable between African American and European populations, and that the literature concerning the prevalence of OSA in Hispanic/Mexican American populations compared with European populations remains inconclusive [47]). 

When analyzed in a multivariate manner, both the statistical and ML model retained age and sex as the most significant sociodemographic parameters.

In the present study, OSA was found to be positively associated with obesity, as was demonstrated in the statistical multivariate analysis, where it had the highest OR (Table 6) as well as in the ML model, which showed that obesity is in the second place in feature importance (Figure 1). This is corroborated by other studies which found obesity as a major risk factor to OSA [26]. Suggested mechanisms for the association were the enlargement of soft tissue structures surrounding the airways, causing them to narrow as well as the indirect effects of obesity causing decreased lung volumes, as well as leptin resistance, both leading to unstable breathing and OSA [26]. An escalation in the prevalence of obesity in the general population has been documented in the recent decades, which may have contributed to the rising prevalence of OSA among adults [48]. 

Unlike age, sex, and obesity, smoking is a less well-established risk factor for OSA. Previous studies reported that smoking is positively associated with higher prevalence of OSA diagnosis [49,50], which was attributed to the consequences of smoking irritation of the throat and subsequent local edema of the upper airways, which might cause upper airways obstruction and OSA [50]. On the other hand, Taveira et al. conducted a meta-analysis that found that after adjusting the results confounding factors there was not enough scientific evidence to confirm the association between OSA and tobacco [51]. This is in line with our findings of a significant association between OSA in the statistical multivariate analysis (Tale 6), but the ML algorithm located smoking only on the 12th place, emphasizing the importance of other parameters as OSA predictors.

In the literature, it was shown that the prevalence of OSA is also increased in patients with a variety of MetS related conditions, including hypertension [52,53], hyperlipidemia [54] total cholesterol, HDL and LDL cholesterol, triglycerides concentrations [55], stroke [56], cardiovascular disease [57], diabetes mellitus (71), CRP [58], and NAFLD [59]. Indeed, MetS has also been associated with inflammation due to oxidative stress caused by obesity and insulin resistance, similarly to OSA which has been linked to inflammation for the same reason [12,60]. This is supported by epidemiological evidence showing that an estimated 50–60% of obese people and patients who have MetS also have OSA, a comorbidity thought to exacerbate MetS’s metabolic, inflammatory, and vascular impairments [26]. A recent study which used a Support Vector Machines (SVM) ML algorithm for identification of OSA severity, demonstrated a higher average impact of dyslipidemia, choking, diabetes, mood disorders, and familiarity for OSA among the independent variable predictors of OSA severity [61]. A review summarized that predictors that warrant screening for OSA include typical symptoms (e.g., snoring, restless sleep, and daytime hyperactivity) or risk factors (e.g., neurologic, genetic, and craniofacial disorders) [62].

In line with these findings, we also found a positive association between OSA and these systemic conditions in the univariate statistical analysis. However, these associations were not retained in the statistical multivariate analysis (Table 6), and the ML algorithm also located these conditions from the sixth place and below in feature importance (Figure 1). This can be explained by the “common risk factor approach” [63] which states that the associations between OSA and these systemic conditions may be explained by shared common profile and risk factors such as age, sex, and obesity, which may retained a significant association in the multivariate statistical and ML models.

About 25 years ago, the discipline of dental sleep medicine was initially defined. During this time, various dental sleep-related conditions such as snoring, obstructive sleep apnea, sleep bruxism, xerostomia, hypersalivation, gastroesophageal reflux disease, and orofacial pain including burning mouth syndrome were identified. Given this, oral healthcare providers are deemed to have a crucial role in preventing, assessing, and managing OSA [64]. One example of why dental healthcare providers need to have knowledge about dental sleep-related conditions is to determine if snoring is linked to the presence of other conditions such as obstructive sleep apnea, sleep bruxism, gastroesophageal reflux disease, xerostomia, and oro-facial pain [65]. OSA may occur due to upper airway obstruction resulting from anatomical and functional abnormalities, such as upper airway collapsibility, particularly those involving the lateral pharyngeal wall (LPW) [66]. Several barbed palatal surgery techniques have been developed over the last twenty years for the treatment of OSA with promising results [67]. Other important associations that should be acknowledged by clinicians are tooth wear, sleep disorders oro-facial pain, oral dryness, GERD, and sleep bruxism [68]. Indeed, sleep disorders are interlinked with each other, which leads to indirect associations as well, and makes the consequences of each single condition difficult to disentangle. Knowledge of these associations is clinically relevant, but more research is needed to confirm their validity [68]. By recognizing these associations, dentists could aid in the early identification and treatment of these conditions [65].

The main and most intriguing of this study’s results is the association between periodontitis and OSA, independent of other parameters that were analyzed. This significant association was found in both the univariate (Table 5) and the multivariate (Table 6) statistical analyses, as well as in the ML model which located periodontitis in the fourth place in feature importance, after the well-known risk factors of age, obesity, and sex (Figure 1). This result is the first of its kind on this large scale, which considered multiple confounders and used both statistical and ML models. Other studies also found a positive association between periodontitis and OSA [13,14,15,16,69], including a recent systematic review and meta-analysis which found that periodontitis has a direct association with OSA [13]. However, there were other studies which found no significant associations between OSA and periodontitis [18,19], and the cause-and-effect relationship has not been found yet, with several possible explanations for the association between OSA and periodontitis were suggested. 

One possible explanation for is that periodontitis causes chronic inflammatory responses in its host which act as a mediator to the OSA inflammatory response or vice versa [15]. Previous studies demonstrated that systemic inflammatory markers such as TNF-α, IL-6, and IL-1B are higher in OSA patients [70]. Moreover, OSA have been shown to alter the tested bacteria in plaque, and OSA correlated with increasing periodontal disease severity, and had an additive effect on salivary IL-6 [71]. 

A second possible explanation is that OSA is associated with mouth breathing, and therefore can increase the risk of periodontitis [15]. OSA has been positively associated with breathing orally and oro-nasally before during sleep [72]. Oral breathing can cause a dry mouth, that can in turn decrease the self-cleaning ability of the oral cavity and as a result may lead to increased bacterial colonization and gingivitis [13]. Therefore, it is possible that OSA can play a role periodontitis pathogenesis through mouth-breathing, and further study into this effect is necessary.

A third possible explanation is that periodontitis and OSA are comorbid as they share many risk factors [15]. These risk factors, such as smoking, obesity, and diabetes, were considered in this present study as possible confounders/mediators, and periodontitis retained a significant positive association with OSA in both the multivariate statistical and ML models, independent of these risk factors. 

All delivered dental procedure that were analyzed had a significant positive association with OSA in the univariate analysis (Table 5), and in the ML model, the delivered dental fillings reached the fifth place and missing teeth reached the seventh place in feature importance (Figure 1). Nevertheless, these associations were less established than the associations between periodontitis and OSA, since periodontitis was in a higher place in feature importance in the ML model (fourth place, Figure 1), and periodontitis retained a statistically significant positive association with OSA in the multivariate analysis, while missing teeth and delivered dental fillings did not (Table 6). 

Previous studies on the relationship between caries and OSA also showed varying results. Pico-Orozco et al. recently found statistically significant association between DMFT scores (decayed, missing and filled teeth) and Apnea–Hypopnea Index (AHI) (which represents OSA severity), but there was no statistically significant association between DMFT scores and the OSA group compared to a control group [21]. Conversely, Acar et al. found no significant association between either OSA or OSA severity (AHI index) and DMFT scores showing no association between OSA and caries experience [22]. 

Possible explanations for the associations were given. The first possibility is through inflammatory mechanisms. Children with OSA had higher DMFT scores, lower mucosal pH scores, worse orthodontic measurements, and higher levels of the inflammatory cytokine IL-1β and salivary Mutans streptococci and Lactobacilli levels [20]. 

A second possible explanation was attributed to the use of devices in the treatment of OSA that were suspected to cause worse dental and oral health. However, there was no significant difference between continuous positive airway pressure (CPAP) or bilevel positive airway pressure (BiPAP) users to controls with no OSA and no CPAP/BiPAP use, regarding dental plaque, calculus, gingival inflammation, number of missing teeth, and masticatory function units [73]. Likewise, the use of removable oral appliances was suspected to worsen the caries experience in OSA patients due to limiting the access of cleansing saliva to the tooth surface, but to this day this hypothesis stayed unsubstantiated [74]. 

### Strengths and Limitations

To the best of our knowledge, this is the first study in the English literature that uses advanced statistical and ML models to study the association between OSA, metabolic dysfunction and dental status using a big data repository of a nationally representative study population (132,529) of young to middle-aged adults. Since Israel is an immigrant’s country, the database included people of different ethnicities and origin countries. A strict protocol and uniform codes were used that were based on doctor diagnoses and auxiliary tests, and not self-reported diagnoses by the patients. The fact that the data were based on record diminishes the impact of patient recall bias, except for health-related habits. The comprehensive DOME repository allowed a very large number of parameters to be examined, from different facets of life: socio-demographical, health-related habits, medical, and dental. Dental and medical care in the IDF is free, which enables us to reach parts of the public that do not get access to dental care or to researchers as often. 

The main limitation of the study is the cross-sectional study design, which cannot suggest cause-and-effect relations. While many confounders were included, due to the complexities of the issues discussed, there are other parameters that were not included such as genetics, microbiome, polysomnography test results and past exposures including childhood exposures.

There is a need for further longitudinal research in different ethnicities and countries, that will take into account more parameters, to determine their causes and origins of the associations found in this study.

## 5. Conclusions

The central hypothesis of this study, that OSA is positively associated with dental morbidity, particularly periodontitis, was supported by these findings. This study suggests a profile of a vulnerable patient of OSA, who is male, obese, is of an older age, and has periodontal disease. The findings highlight the need for dental evaluation as part of the workup of OSA patients and emphasizes the need for dental and general medical authorities to collaborate by exchanging knowledge about dental and systemic morbidities and their associations. By recognizing these associations, dentists and physicians could aid in the early identification and treatment of OSA and its related dental and systemic conditions. The study also highlights the necessity for a comprehensive holistic risk management strategy that takes systemic and dental diseases into account.

## Figures and Tables

**Table 1:** Socio-demographic characteristics according to obstructive sleep apnea (OSA) diagnosis * Pearson Chi-Square; ^ likelihood ratio; # binary logistic regression; ** non-paired *t*-test; ## generalized linear models. OR—odds ratio, CI—confidence interval, SD—standard deviation.

Parameters	Variables	Without OSA	OSA	*p* Value	OR (95% CI) #
		No. (%) %	No. (%) %		
Sex	Women	33,045 (99.9)	18 (0.1)	<0.001 *	1
	Men	99,166 (99.7)	300 (0.3)		5.52 (3.45–8.93)
Educational level	High school	112,956 (100)	56 (0.0)	<0.001 ^	0.04 (0.03–0.05)
	Technicians	7319 (98.6)	107 (1.4)		1.19 (0.93–1.53)
	Academic	12,661 (98.8)	155 (1.2)		1
Residency location	Urban non-Jewish	17,887 (99.8)	31 (0.2)	0.002 ^	0.70 (0.48–1.02)
	Rural	577 (99.0)	6 (1.0)		4.20 (1.86–9.48)
	Urban Jewish	113,188 (99.8)	280 (0.2)		1
Socio-economic status (SES)	Low	5710 (99.8)	9 (0.2)	0.270 ^	1
	Medium	68,460 (99.8)	159 (0.2)		1.47 (0.75–2.89)
	High	56,562 (99.7)	145 (0.3)		1.63 (0.83–3.19)
Rings of a city/town	Central rings—midtown	118,164 (99.8)	286 (0.2)	0.745 *	1.06 (0.74–1.53)
	Peripheral rings—suburbs	14,047 (99.8)	32 (0.2)		1
Birth country	Western Europe	10,538 (99.7)	33 (0.3)	0.096 ^	1.34 (0.93–1.92)
	Eastern Europe	1708 (99.6)	7 (0.4)		1.75 (0.82–3.71)
	Asia	505 (99.2)	4 (0.8)		3.38 (0.25–9.11)
	Africa Ethiopia	2527 (99.99)	3 (0.001)		0.59 (0.19–1.83)
	North America	2855 (99.9)	4 (0.1)		0.60 (0.22–1.61)
	South America	955 (99.8)	2 (0.2)		0.89 (0.22–3.60)
	Israel	113,094 (99.8)	265 (0.2)		1
Parameter	OSA	Mean ± SD		*p* value **	OR (95% CI) ##
Age	No	21.85 ± 5.97		<0.001	1.19 (1.18–1.21)
	Yes	37.38 ± 8.08			
Length of service	No	3.09 ± 6.23		<0.001	1.09 (1.08–1.10)
	Yes	19.19 ± 9.61			

**Table 2:** Health related risk factors and medical and dental attendance patterns according to OSA * Pearson Chi-Square; ** Binary logistic regression.

Parameter	Variable	Without OSA	OSA	*p* Value *	OR (95% CI) **
		No. (%) %	No. (%) %		
Current smoking	No	125,445 (99.8)	200 (0.2)	<0.001	1
	Yes	6766 (98.3)	118 (1.7)		10.75 (8.62–13.51)
Teeth brushing at least once a day or more	No	17,736 (99.5)	84 (0.5)	<0.001	1
	Yes	39,573 (99.7)	103 (0.3)		0.54 (0.41–0.73)
Parameter	OSA	Mean ± SD		*p* * value *	OR (95% CI) **
Total number of dental appointments	No	5.86 ± 10.21		<0.001	1.03 (1.02–1.03)
	Yes	16.17 ± 19.84			
Non-attendance to scheduled dental appointments	No	0.99 ± 2.66		<0.001	1.06 (1.05–1.08)
	Yes	2.33 ± 4.46			
Total number of appointments with a general physician	No	14.21 ± 11.98		<0.001	1.02 (1.01–1.03)
	Yes	18.09 ± 15.86			

**Table 3:** Metabolic morbidity according to OSA * Pearson Chi-Square; ** Binary logistic regression.

Parameter	Variable	Without OSA	OSA	*p* * Value	OR (95% CI) **
		No. (%) %	No. (%) %		
Hypertension	No	128,906 (99.8)	260 (0.2)	<0.001	1
	Yes	3305 (98.3)	58 (1.7)		8.55 (6.45–11.36)
Hyperlipidemia	No	131,263 (99.8)	305 (0.2)	<0.001	1
	Yes	948 (98.6)	13 (1.4)		5.85 (3.37–10.10)
Diabetes Mellitus	No	131,880 (99.8)	304 (0.2)	<0.001	1
	Yes	331 (95.9)	14 (4.1)		17.54 (10.42–29.41)
Obesity	No	124,949 (99.9)	132 (0.1)	<0.001	1
	Yes	7262 (97.5)	186 (2.5)		23.81 (18.87–29.41)
Cardiovascular disease	No	128,669 (99.8)	262 (0.2)	<0.001	1
	Yes	3542 (98.4)	56 (1.6)		7.63 (5.75–10.20)
Non-alcoholic fatty liver disease (NAFLD)	No	131,308 (99.8)	283 (0.2)	<0.001	1
	Yes	903 (96.3)	35 (3.7)		17.24 (12.34–24.39)
Stroke	No	132,121 (99.8)	316 (0.2)	<0.001	1
	Yes	90 (97.8)	2 (2.2)		9.09 (2.30–35.71)
Transient ischemic attack (TIA)	No	132,114 (99.8)	316 (0.2)	<0.001	1
	Yes	97 (98.0)	2 (2.0)		8.47 (2.14–33.33)

**Table 4:** Medical indices according to OSA (* non-paired *t* test, ** generalized linear models).

Parameter	OSA	N	Mean ± SD	*p* Value *	OR (95% CI) **
Weight (kilograms)	No	66,371	73.23 ± 32.41	<0.001	1.004 (1.003–1.005)
	Yes	246	90.65 ± 18.14		
Body mass index (BMI)	No	66,149	24.26 ± 4.29	<0.001	1.188 (1.165–1.212)
	Yes	245	29.17 ± 5.00		
C-reactive protein (CRP) (mg/L)	No	30,269	3.76 ± 10.16	0.007	1.011 (1.003–1.019)
	Yes	149	6.02 ± 14.19		
Cholesterol (mg/dL)	No	27,895	175.76 ± 35.62	<0.001	1.005 (1.002–1.008)
	Yes	285	182.60 ± 36.47		
High-density lipoprotein (HDL) (mg/dL)	No	27,888	48.33 ± 11.79	<0.001	0.958 (0.947–0.970)
	Yes	285	43.54 ± 9.17		
Low-density lipoprotein (LDL) (mg/dL)	No	19,945	108.28 ± 29.99	0.012	1.005 (1.001–1.009)
	Yes	268	112.92 ± 32.83		
LDL cholesterol calculated (mg/dL)	No	17,254	108.31 ± 30.41	0.018	1.005 (1.001–1.010)
	Yes	204	113.38 ± 30.46		
Non-HDL cholesterol (mg/dL)	No	16,588	129.37 ± 34.98	<0.001	1.007 (1.004–1.011)
	Yes	234	139.11 ± 35.77		
Very-low-density lipoprotein (VLDL) (mg/dL)	No	27,846	20.56 ± 11.17	<0.001	1.030 (1.022–1.038)
	Yes	285	25.64 ± 13.29		
Triglycerides	No	27,898	104.23 ± 63.97	<0.001	1.003 (1.002–1.004)
	Yes	285	128.20 ± 66.38		
Glycated hemoglobin (HbA1c) (%)	No	1896	5.40 ± 0.97	0.092	1.207 (0.967–1.505)
	Yes	47	5.64 ± 0.98		
Fasting blood glucose (mg/dL)	No	2486	87.10 ± 11.97	0.369	1.010 (0.989–1.032)
	Yes	41	88.79 ± 9.90		

**Table 5:** The association of OSA with periodontitis and with planned and delivered dental procedures among the study population * non-paired *t* test, ** Generalized Linear Models.

Parameter	Variable	No OSA	OSA	*p* * Value	OR (95% CI) **
		No. (%)	No. (%)		
Periodontal Disease	No	51,730 (99.7)	136 (0.3)	<0.001	1
	Yes	5579 (99.1)	51 (0.9)		3.46 (2.51–4.76)
The number of delivered dental procedures		Without OSA (Mean ± SD)	OSA (Mean ± SD)	*p* value *	OR (95% confidence interval) **
Fillings		1.01 ± 1.97	1.27 ± 1.91	0.018	1.05 (1.01–1.10)
Endodontic treatments		0.08 ± 0.34	0.16 ± 0.50	0.004	1.43 (1.22–1.68)
Renewal of endodontic treatment		0.02 ± 0.17	0.08 ± 0.35	<0.001	1.43 (1.18–1.72)
Regular extractions		0.10 ± 0.43	0.20 ± 0.51	<0.001	1.19 (1.09–1.30)
Surgical extractions		0.05 ± 0.28	0.09 ± 0.32	0.035	1.37 (1.07–1.75)
Indirect post and core		0.10 ± 0.40	0.23 ± 0.59	<0.001	1.38 (1.23–1.54)
Direct post and core		0.00 ± 0.09	0.04 ± 0.29	0.014	1.59 (1.29–1.95)
Crowns		0.05 ± 0.54	0.40 ± 1.74	<0.001	1.25 (1.04–1.45)
The number of missing teeth		0.58 ± 1.29	1.21 ± 1.67	<0.001	1.13 (1.09–1.18)

**Table 6:** Multivariate analysis and collinearity statistics with OSA as a dependent variable with statistically significant parameters in the univariate analysis * binary logistic regression, ** linear regression, VIF: variance inflation factor.

		Multivariate Binary Logistic Regression Analysis *				Collinearity Statistics Using Linear Regression Analysis **	
Parameter		B	Std. Error	*p* Value	OR (95% CI) *	Tolerance	VIF
(Intercept)		9.845	0.738	<0.001	0.002 (0.001–0.004)		
Age		0.134	0.010	<0.001	1.14 (1.12–1.17)	0.509	1.966
Sex: men/women		0.880	0.333	0.008	2.41 (1.25–4.63)	0.955	1.047
Residency location	Urban Jewish	−0.178	0.519	0.731	0.84 (0.30–2.32)	0.979	1.021
	Urban non-Jewish	−0.443	0.563	0.432	0.64 (0.21–1.94)	0.994	1.006
Smoking		0.372	0.162	0.022	1.45 (1.05–1.99)	0.760	1.317
Teeth brushing once a day or more		−0.195	0.180	0.279	0.82 (0.58–1.17)	0.738	1.355
Hypertension		0.358	0.202	0.077	1.43 (0.96–2.13)	0.910	1.099
Hyperlipidemia		0.971	0.512	0.058	2.63 (0.96–7.19)	0.958	1.044
Diabetes Mellitus		0.112	0.393	0.776	1.12 (0.52–2.42)	0.948	1.054
Obesity		1.133	0.181	<0.001	3.10 (2.18–4.42)	0.690	1.449
Cardiovascular disease		0.005	0.213	0.983	1.00 (0.66–1.53)	0.932	1.073
Non-alcoholic fatty liver disease (NAFLD)		0.091	0.266	0.733	1.09 (0.65–1.84)	0.905	1.105
Stroke		0.029	1.054	0.978	1.03 (0.13–8.13)	0.939	1.065
Transient ischemic attack (TIA)		0.548	1.047	0.601	1.73 (0.22–13.5)	0.937	1.067
Total number of dental appointments		0.003	0.005	0.618	1.00 (0.99–1.01)	0.468	2.136
Non-attendance to scheduled dental appointments		0.013	0.023	0.558	1.01 (0.96–1.06)	0.526	1.901
Periodontal Disease		0.699	0.189	<0.001	2.01 (1.38–2.91)	0.967	1.034
Missing teeth		−0.057	0.053	0.282	0.94 (0.85–1.05)	0.800	1.250
Delivered dental fillings		−0.062	0.037	0.100	0.94 (0.87–1.01)	0.929	1.077

## Data Availability

Data sharing not applicable during public privacy.

## References

[1] Jordan A.S., McSharry D.G., Malhotra A. (2014). Adult obstructive sleep apnoea. Lancet.

[2] Veasey S.C., Rosen I.M. (2019). Obstructive Sleep Apnea in Adults. N. Engl. J. Med..

[3] Chang H.P., Chen Y.F., Du J.K. (2020). Obstructive sleep apnea treatment in adults. Kaohsiung J. Med. Sci..

[4] Yaggi H.K., Strohl K.P. (2010). Adult obstructive sleep apnea/hypopnea syndrome: Definitions, risk factors, and pathogenesis. Clin. Chest Med..

[5] Salari N., Khazaie H., Abolfathi M., Ghasemi H., Shabani S., Rasoulpoor S., Mohammadi M., Rasoulpoor S., Khaledi-Paveh B. (2022). The effect of obstructive sleep apnea on the increased risk of cardiovascular disease: A systematic review and meta-analysis. Neurol. Sci..

[6] Mitra A.K., Bhuiyan A.R., Jones E.A. (2021). Association and Risk Factors for Obstructive Sleep Apnea and Cardiovascular Diseases: A Systematic Review. Diseases.

[7] Falkner B., Cossrow N.D. (2014). Prevalence of metabolic syndrome and obesity-associated hypertension in the racial ethnic minorities of the United States. Curr. Hypertens. Rep..

[8] Sanz M., Herrera D., Kebschull M., Chapple I., Jepsen S., Beglundh T., Sculean A., Tonetti M.S., Participants E.F.P.W., Methodological C. (2020). Treatment of stage I-III periodontitis-The EFP S3 level clinical practice guideline. J. Clin. Periodontol..

[9] Papapanou P.N., Sanz M., Buduneli N., Dietrich T., Feres M., Fine D.H., Flemmig T.F., Garcia R., Giannobile W.V., Graziani F. (2018). Periodontitis: Consensus report of workgroup 2 of the 2017 World Workshop on the Classification of Periodontal and Peri-Implant Diseases and Conditions. J. Periodontol..

[10] Kwon T., Lamster I.B., Levin L. (2021). Current Concepts in the Management of Periodontitis. Int. Dent. J..

[11] Grigalauskiene R., Slabsinskiene E., Vasiliauskiene I. (2015). Biological approach of dental caries management. Stomatologija.

[12] Arnaud C., Bochaton T., Pepin J.L., Belaidi E. (2020). Obstructive sleep apnoea and cardiovascular consequences: Pathophysiological mechanisms. Arch. Cardiovasc. Dis..

[13] Khodadadi N., Khodadadi M., Zamani M. (2022). Is periodontitis associated with obstructive sleep apnea? A systematic review and meta-analysis. J. Clin. Exp. Dent..

[14] Al-Jewair T.S., Al-Jasser R., Almas K. (2015). Periodontitis and obstructive sleep apnea’s bidirectional relationship: A systematic review and meta-analysis. Sleep Breath..

[15] Gunaratnam K., Taylor B., Curtis B., Cistulli P. (2009). Obstructive sleep apnoea and periodontitis: A novel association?. Sleep Breath..

[16] Zhang Z., Ge S., Zhai G., Yu S., Cui Z., Si S., Chou X. (2022). Incidence and risk of periodontitis in obstructive sleep apnea: A meta-analysis. PLoS ONE.

[17] Lembo D., Caroccia F., Lopes C., Moscagiuri F., Sinjari B., D’Attilio M. (2021). Obstructive Sleep Apnea and Periodontal Disease: A Systematic Review. Medicina.

[18] Loke W., Girvan T., Ingmundson P., Verrett R., Schoolfield J., Mealey B.L. (2015). Investigating the association between obstructive sleep apnea and periodontitis. J. Periodontol..

[19] Sales-Peres S.H., Groppo F.C., Rojas L.V., de Sales-Peres C.M., Sales-Peres A. (2016). Periodontal Status in Morbidly Obese Patients With and Without Obstructive Sleep Apnea Syndrome Risk: A Cross-Sectional Study. J. Periodontol..

[20] Davidovich E., Hevroni A., Gadassi L.T., Spierer-Weil A., Yitschaky O., Polak D. (2022). Dental, oral pH, orthodontic and salivary values in children with obstructive sleep apnea. Clin. Oral. Investig..

[21] Pico-Orozco J., Silvestre F.J., Carrasco-Llatas M., Silvestre-Rangil J. (2022). Dental caries status in adults with sleep apnea-hypopnea syndrome. J. Clin. Exp. Dent..

[22] Acar M., Turkcan I., Ozdas T., Bal C., Cingi C. (2015). Obstructive sleep apnoea syndrome does not negatively affect oral and dental health. J. Laryngol. Otol..

[23] Kang I.A., Njimbouom S.N., Kim J.D. (2023). Optimal Feature Selection-Based Dental Caries Prediction Model Using Machine Learning for Decision Support System. Bioengineering.

[24] Ertas K., Pence I., Cesmeli M.S., Ay Z.Y. (2023). Determination of the stage and grade of periodontitis according to the current classification of periodontal and peri-implant diseases and conditions (2018) using machine learning algorithms. J. Periodontal Implant. Sci..

[25] Ben-Assuli O., Bar O., Geva G., Siri S., Tzur D., Almoznino G. (2022). Body Mass Index and Caries: Machine Learning and Statistical Analytics of the Dental, Oral, Medical Epidemiological (DOME) Nationwide Big Data Study. Metabolites.

[26] Drager L.F., Togeiro S.M., Polotsky V.Y., Lorenzi-Filho G. (2013). Obstructive sleep apnea: A cardiometabolic risk in obesity and the metabolic syndrome. J. Am. Coll. Cardiol..

[27] Fietze I., Laharnar N., Obst A., Ewert R., Felix S.B., Garcia C., Glaser S., Glos M., Schmidt C.O., Stubbe B. (2019). Prevalence and association analysis of obstructive sleep apnea with gender and age differences—Results of SHIP-Trend. J. Sleep Res..

[28] Almoznino G., Kedem R., Turgeman R., Bader T., Yavnai N., Zur D., Shay B. (2020). The Dental, Oral, Medical Epidemiological (DOME) Study: Protocol and Study Methods. Methods Inf. Med..

[29] Abramovitz I., Zini A., Atzmoni M., Kedem R., Zur D., Protter N.E., Almoznino G. (2021). Cognitive Performance and Its Associations with Dental Caries: Results from the Dental, Oral, Medical Epidemiological (DOME) Records-Based Nationwide Study. Biology.

[30] Abramovitz I., Zini A., Kessler Baruch O., Kedem R., Protter N.E., Shay B., Yavnai N., Zur D., Mijiritsky E., Almoznino G. (2021). SOS teeth with advanced caries and sociodemographic indicators, health-related habits and dental attendance patterns: Data from the Dental, Oral, Medical Epidemiological (DOME) nationwide records-based study. BMC Oral. Health.

[31] Abramovitz I., Zini A., Pribluda P., Kedem R., Zur D., Protter N.E., Almoznino G. (2021). “Dental Cluster” Versus “Metabolic Cluster”: Analyzing the Associations of Planned and Delivered Dental Procedures with Metabolic Syndrome, Utilizing Data from the Dental, Oral, Medical Epidemiological (DOME) Cross-Sectional Record-Based Nationwide Study. Biology.

[32] Almoznino G., Kessler Baruch O., Kedem R., Protter N.E., Shay B., Yavnai N., Zur D., Mijiritsky E., Abramovitz I. (2020). SOS Teeth: First Priority Teeth with Advanced Caries and Its Associations with Metabolic Syndrome among a National Representative Sample of Young and Middle-Aged Adults. J. Clin. Med..

[33] Almoznino G., Zini A., Kedem R., Protter N.E., Zur D., Abramovitz I. (2021). Hypertension and Its Associations with Dental Status: Data from the Dental, Oral, Medical Epidemiological (DOME) Nationwide Records-Based Study. J. Clin. Med..

[34] Ram D., Wilensky A., Zur D., Almoznino G. (2022). The Triangle of Nonalcoholic Fatty Liver Disease, Metabolic Dysfunction, and Periodontitis: Analysis of the Dental, Oral, Medical and Epidemiological (DOME) Records-Based Nationwide Research. Metabolites.

[35] American Academy of Sleep Medicine (2014). International Classification of Sleep Disorders.

[36] Pedregosa F., Varoquaux G., Gramfort A., Michel V., Thirion B., Grisel O., Blondel M., Prettenhofer P., Weiss R., Dubourg V. (2011). Scikit-Learn: Machine Learning in Python. J. Mach. Learn. Res..

[37] Friedman J. (2001). Greedy function approximation: A gradient boosting machine. Ann. Statist..

[38] Liu H.Z.M., Lu X.S., Yao C. Weighted Gini index feature selection method for imbalanced data. Proceedings of the ICNSC 2018—15th IEEE International Conference on Networking, Sensing and Control.

[39] Huang N., Lu G., Cai G., Xu D., Xu J., Li F., Zhang L. (2016). Feature selection of power quality disturbance signals with an entropy-importance-based random forest. Entropy.

[40] Senaratna C.V., Perret J.L., Lodge C.J., Lowe A.J., Campbell B.E., Matheson M.C., Hamilton G.S., Dharmage S.C. (2017). Prevalence of obstructive sleep apnea in the general population: A systematic review. Sleep Med. Rev..

[41] Tufik S., Santos-Silva R., Taddei J.A., Bittencourt L.R. (2010). Obstructive sleep apnea syndrome in the Sao Paulo Epidemiologic Sleep Study. Sleep Med..

[42] Heinzer R., Marti-Soler H., Marques-Vidal P., Tobback N., Andries D., Waeber G., Preisig M., Vollenweider P., Haba-Rubio J. (2018). Impact of sex and menopausal status on the prevalence, clinical presentation, and comorbidities of sleep-disordered breathing. Sleep Med..

[43] Punjabi N.M. (2008). The epidemiology of adult obstructive sleep apnea. Proc. Am. Thorac. Soc..

[44] Papadopoulos D., Kikemeni A., Skourti A., Amfilochiou A. (2018). The influence of socio-economic status on the severity of obstructive sleep apnea: A cross-sectional observational study. Sleep Sci..

[45] Guglielmi O., Lanteri P., Garbarino S. (2019). Association between socioeconomic status, belonging to an ethnic minority and obstructive sleep apnea: A systematic review of the literature. Sleep Med..

[46] Etindele Sosso F.A., Matos E. (2021). Socioeconomic disparities in obstructive sleep apnea: A systematic review of empirical research. Sleep Breath..

[47] Hnin K., Mukherjee S., Antic N.A., Catcheside P., Chai-Coetzer C.L., McEvoy D., Vakulin A. (2018). The impact of ethnicity on the prevalence and severity of obstructive sleep apnea. Sleep Med. Rev..

[48] Peppard P.E., Young T., Barnet J.H., Palta M., Hagen E.W., Hla K.M. (2013). Increased prevalence of sleep-disordered breathing in adults. Am. J. Epidemiol..

[49] Esen A.D., Akpinar M. (2021). Relevance of obstructive sleep apnea and smoking: Obstructive sleep apnea and smoking. Fam. Pract..

[50] Bielicki P., Trojnar A., Sobieraj P., Wasik M. (2019). Smoking status in relation to obstructive sleep apnea severity (OSA) and cardiovascular comorbidity in patients with newly diagnosed OSA. Adv. Respir. Med..

[51] Taveira K.V.M., Kuntze M.M., Berretta F., de Souza B.D.M., Godolfim L.R., Demathe T., De Luca Canto G., Porporatti A.L. (2018). Association between obstructive sleep apnea and alcohol, caffeine and tobacco: A meta-analysis. J. Oral. Rehabil..

[52] Hou H., Zhao Y., Yu W., Dong H., Xue X., Ding J., Xing W., Wang W. (2018). Association of obstructive sleep apnea with hypertension: A systematic review and meta-analysis. J. Glob. Health.

[53] Ahmad M., Makati D., Akbar S. (2017). Review of and Updates on Hypertension in Obstructive Sleep Apnea. Int. J. Hypertens..

[54] Gunduz C., Basoglu O.K., Hedner J., Bonsignore M.R., Hein H., Staats R., Bouloukaki I., Roisman G., Pataka A., Sliwinski P. (2019). Hyperlipidaemia prevalence and cholesterol control in obstructive sleep apnoea: Data from the European sleep apnea database (ESADA). J. Intern. Med..

[55] Gunduz C., Basoglu O.K., Hedner J., Zou D., Bonsignore M.R., Hein H., Staats R., Pataka A., Barbe F., Sliwinski P. (2018). Obstructive sleep apnoea independently predicts lipid levels: Data from the European Sleep Apnea Database. Respirology.

[56] Yaggi H.K., Concato J., Kernan W.N., Lichtman J.H., Brass L.M., Mohsenin V. (2005). Obstructive sleep apnea as a risk factor for stroke and death. N. Engl. J. Med..

[57] Yeghiazarians Y., Jneid H., Tietjens J.R., Redline S., Brown D.L., El-Sherif N., Mehra R., Bozkurt B., Ndumele C.E., Somers V.K. (2021). Obstructive Sleep Apnea and Cardiovascular Disease: A Scientific Statement From the American Heart Association. Circulation.

[58] Imani M.M., Sadeghi M., Farokhzadeh F., Khazaie H., Brand S., Dursteler K.M., Bruhl A., Sadeghi-Bahmani D. (2021). Evaluation of Blood Levels of C-Reactive Protein Marker in Obstructive Sleep Apnea: A Systematic Review, Meta-Analysis and Meta-Regression. Life.

[59] Jin S., Jiang S., Hu A. (2018). Association between obstructive sleep apnea and non-alcoholic fatty liver disease: A systematic review and meta-analysis. Sleep Breath..

[60] Rochlani Y., Pothineni N.V., Kovelamudi S., Mehta J.L. (2017). Metabolic syndrome: Pathophysiology, management, and modulation by natural compounds. Ther. Adv. Cardiovasc. Dis..

[61] Maniaci A., Riela P.M., Iannella G., Lechien J.R., La Mantia I., De Vincentiis M., Cammaroto G., Calvo-Henriquez C., Di Luca M., Chiesa Estomba C. (2023). Machine Learning Identification of Obstructive Sleep Apnea Severity through the Patient Clinical Features: A Retrospective Study. Life.

[62] Bitners A.C., Arens R. (2020). Evaluation and Management of Children with Obstructive Sleep Apnea Syndrome. Lung.

[63] Sheiham A., Watt R.G. (2000). The common risk factor approach: A rational basis for promoting oral health. Community Dent. Oral. Epidemiol..

[64] Huang Z., Zhou N., Lobbezoo F., Almeida F.R., Cistulli P.A., Dieltjens M., Huynh N.T., Kato T., Lavigne G.J., Masse J.F. (2023). Dental sleep-related conditions and the role of oral healthcare providers: A scoping review. Sleep Med. Rev..

[65] Huang Z., Zhou N., Chattrattrai T., van Selms M.K.A., de Vries R., Hilgevoord A.A.J., de Vries N., Aarab G., Lobbezoo F. (2023). Associations between snoring and dental sleep conditions: A systematic review. J. Oral. Rehabil..

[66] Cammaroto G., Stringa L.M., Iannella G., Meccariello G., Zhang H., Bahgat A.Y., Calvo-Henriquez C., Chiesa-Estomba C., Lechien J.R., Barillari M.R. (2020). Manipulation of Lateral Pharyngeal Wall Muscles in Sleep Surgery: A Review of the Literature. Int. J. Environ. Res. Public Health.

[67] Gulotta G., Iannella G., Meccariello G., Cammaroto G., Visconti I.C., de Vincentiis M., Greco A., Pelucchi S., Magliulo G., Ruoppolo G. (2021). Barbed suture Extrusion and Exposure in palatoplasty for OSA: What does it mean?. Am. J. Otolaryngol..

[68] Wetselaar P., Manfredini D., Ahlberg J., Johansson A., Aarab G., Papagianni C.E., Reyes Sevilla M., Koutris M., Lobbezoo F. (2019). Associations between tooth wear and dental sleep disorders: A narrative overview. J. Oral. Rehabil..

[69] Mukherjee S., Galgali S.R. (2021). Obstructive sleep apnea and periodontitis: A cross-sectional study. Indian. J. Dent. Res..

[70] Nadeem R., Molnar J., Madbouly E.M., Nida M., Aggarwal S., Sajid H., Naseem J., Loomba R. (2013). Serum inflammatory markers in obstructive sleep apnea: A meta-analysis. J. Clin. Sleep Med..

[71] Nizam N., Basoglu O.K., Tasbakan M.S., Lappin D.F., Buduneli N. (2016). Is there an association between obstructive sleep apnea syndrome and periodontal inflammation?. Clin. Oral. Investig..

[72] Koutsourelakis I., Vagiakis E., Roussos C., Zakynthinos S. (2006). Obstructive sleep apnoea and oral breathing in patients free of nasal obstruction. Eur. Respir. J..

[73] Carra M.C., Thomas F., Schmitt A., Pannier B., Danchin N., Bouchard P. (2016). Oral health in patients treated by positive airway pressure for obstructive sleep apnea: A population-based case-control study. Sleep Breath..

[74] Dioguardi A., Al-Halawani M. (2016). Oral Appliances in Obstructive Sleep Apnea. Otolaryngol. Clin. N. Am..

